# Cerebellar Hemorrhage and Spinal Fluid Overdrainage With Tonsillar Herniation Following Spine Surgery

**DOI:** 10.7759/cureus.10418

**Published:** 2020-09-13

**Authors:** Sudhakar Kinthala, Kuiran Jiao, Abistanand Ankam, Christopher G Paramore

**Affiliations:** 1 Anesthesiology, Guthrie Robert Packer Hospital, Sayre, USA; 2 Neurological Surgery, Guthrie Robert Packer Hospital, Sayre, USA

**Keywords:** remote cerebellar hemorrhage, csf, overdrainage, spine surgery, durotomy, tonsillar herniation, case report, ventriculostomy, postoperative complication, postoperative

## Abstract

Spinal fluid overdrainage with cerebellar hemorrhage is a rare complication of spinal surgery that can have severe consequences if not detected quickly. We present the case of a 72-year-old Caucasian female who underwent thoracolumbar fixation for flatback syndrome. Intraoperatively, the patient suffered a dural injury that was repaired. In the immediate postoperative period, the patient’s neurological status rapidly deteriorated within an hour and Jackson-Pratt (JP) drain output measured 300 ml of serosanguinous fluid. A stat CT scan revealed cerebellar hemorrhage, pneumocephalus, and tonsillar herniation. The postoperative drain was immediately removed, and a ventriculostomy tube was placed, confirming low intracranial pressure. Postoperatively, the patient was electively ventilated for three days, continued with remote cerebellar hemorrhage (RCH) treatment and precaution, and extubated on the third day as the patient’s neurological function continued to improve. The patient was discharged home nine days after the initial surgery, with a complete recovery. This case indicates that wound drainage in the face of durotomy can induce cerebellar herniation as early as within an hour postoperatively following spine surgery with a dural tear, even after dural repair. This case also suggests that early recognition and appropriate management of RCH is the key to a full recovery. Even in the event of tonsillar herniation and cerebellar hemorrhage, a complete recovery is possible with early recognition and proper management.

## Introduction

Tonsillar herniation associated with remote cerebellar hemorrhage (RCH), which develops distant to the site of surgery, is a rare but potentially lethal complication of supratentorial craniotomy or spinal surgery [1]. Chadduck first described an RCH after a cervical laminectomy with cerebrospinal fluid (CSF) loss in 1981 [2]. Cevik et al. found that the incidence rate of RCH in more than 2,000 lumbar procedures was 0.08% [3]. Floman et al. observed an incidence rate of 0.26% for RCH in their study of 210 patients who had CSF loss following lumbar spine surgery [4]. RCH has a variable timeline of presentation. Konya et al. reviewed the published literature up to 2006 and noted that an RCH was diagnosed between 16 and 120 hours after surgery (47 hours on average) [5,6]. Although RCH was believed to be a delayed complication, a few recent cases of an RCH that presented prior to extubation [7] or in the immediate postoperative period (first one hour) have been reported [8-10]; however, none of them developed cerebellar herniation in the immediate postoperative period.

To the best of our knowledge, this is the first case where the patient developed cerebellar herniation, associated with RCH and pneumocephalus in the first hour following spine surgery. More importantly, with early recognition and proper management, the patient was able to completely recover from this extreme event.

## Case presentation

A 72-year-old Caucasian female, with a body mass index of 29, with a past medical history of hypertension and American Society of Anesthesiology grade (ASA) II, was diagnosed with the flat back syndrome. Following failed medical management, the patient was scheduled for a posterior osteotomy with interbody fusion of the L2-3, L3-4, and L5-S1 vertebra using interbody cages and autograft, posterior lateral fusion at L1-2 utilizing allograft, and posterior segmental fixation from T11- S1 with S2 pelvic instrumentation utilizing neuronavigation. Under the standard ASA monitoring, arterial line placement, and electrophysiological neuromonitoring, the patient received general anesthesia with an endotracheal tube. Induction, intubation, and positioning were uneventful. Anesthesia was maintained with total intravenous anesthesia with remifentanil and propofol to facilitate electrophysiological neuromonitoring of the spine. Intraoperative O arm and neuronavigation were used for instrumentation guidance. The duration of surgery and anesthesia was 6.5 and 7.5 hours, respectively. The total intraoperative fluid input was 5.5 L of crystalloid. The estimated blood loss was 500 mL and urine output was 700 mL. Other than an accidental iatrogenic dural tear at the L3 level, which was repaired with multiple sutures, there were no intraoperative critical events. The patient was extubated in the operating room upon meeting the standard extubation criteria. The extubation was smooth and not associated with any coughing or bucking.

**Figure 1:**
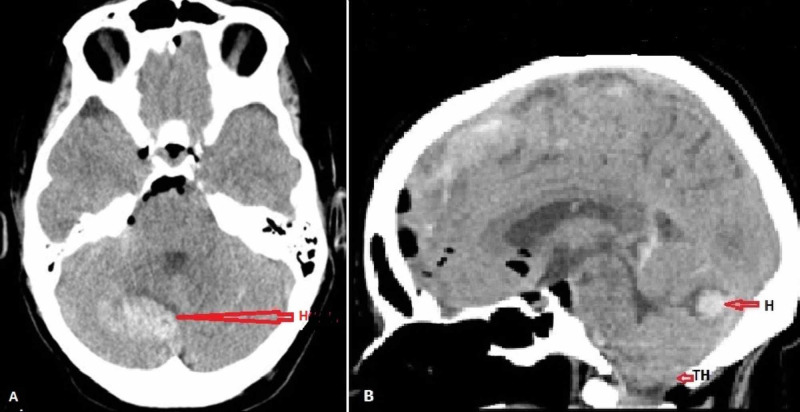
(A) CT axial section showing cerebellar hemorrhage (H). (B) CT sagittal section showing cerebellar hemorrhage (H) and tonsillar herniation (TH).

Postoperatively, the patient progressively became unarousable, Glasgow Coma Scale (GCS) 9-10, with labored breathing, requiring bilevel positive airway pressure (BiPAP). The pupils were still reacting to light though sluggish, associated with hypertension and tachycardia. A stat CT scan was ordered to rule out an intracranial process. By the time the CT scan was completed, the patient’s eyes were rolling downward, pupils were dilated with no reaction to light. Meanwhile, the Jackson-Pratt (JP) drain from the surgical site drained out approximately 300 mL of blood-tinged fluid over the past one hour following surgery. The head CT scan revealed the right cerebellar hemorrhage (Figure 1A), pneumocephalus, and herniated cerebellar tonsils at the foramen magnum (Figure 1B). The JP drain was removed immediately. The patient was intubated and a ventriculostomy was placed, which confirmed low intracranial pressure (ICP). Intraoperatively, the patient was started on a phenylephrine infusion to support her blood pressure; otherwise, there were no intraoperative events.

Postoperatively, the patient was transported to the intensive care unit for elective ventilation. The patient remained in the supine position postoperatively, and the ventriculostomy tube was clamped off. On the first postoperative day, the patient was still intubated and unarousable. However, she was breathing spontaneously, able to move the right upper and both lower extremities randomly, but her eyes were still rolling downwards. The phenylephrine infusion was stopped. Over the next two days, the patient’s neurological function continued to improve. While maintaining in the supine position for most of the time, the patient’s head was gently raised to 10 degrees for a short period of time every day. On the third day, the patient was awake and alert, following commands, moving all her extremities with good strength, pupils reacted to light briskly, and was extubated on day 3. The patient had a complete neurological recovery and was discharged home on day 9 after the initial surgery. At a follow-up visit one month, the patient was doing well, having an excellent functional and neurological status, with no residual complication.

## Discussion

Overdrainange of spinal fluid associated with tonsillar herniation and cerebellar hemorrhage is a potentially lethal complication of supratentorial craniotomy and spinal surgery [11], Sturiale et al. reviewed 57 cases of RCH and found that intraoperative dural lesions were described in approximately 93% of patients. Coagulation disorders, hypertension, and placement of postoperative subfascial drainages were the most frequently reported risk factors for the development of RCH [1]. The most likely mechanism of RCH development is intraoperative or postoperative CSF leakage, resulting in excessive CSF drainage and downward displacement and stretching/occlusion of the cerebellar veins resulting in a hemorrhagic venous infarction [11]. Even with tight closure of the dural wound, an excessive CSF leak can occur and potentially result in an RCH and tonsillar herniation [12].

Most of the available literature mentions that RCH develops as a delayed complication of spinal surgery. The clinical presentation ranges from headache, drowsiness, deterioration in consciousness, features of transient cerebellar, and brainstem dysfunction to a large hematoma, causing obstructive hydrocephalus [11,13,14]. However, an RCH should be suspected in any patient with a delayed emergence from anesthesia, following spine surgery complicated by a dural tear and CSF leak, unexplained deterioration of consciousness, and postoperative neurological changes [13,14]. If an RCH is suspected, an emergency CT scan should be performed because of potential cerebellar herniation, and any delay could lead to significant neurological damage. If the patient is clinically stable, a myelogram could be performed to diagnose CSF leak, and an epidural blood patch could be placed to prevent further CSF leak [12].

In our case, despite an intraoperative durotomy that was repaired, the use of a JP drain led to excessive CSF drainage (300 mL within an hour) leak and rapid ICP drop. When a pressure gradient was developed across the foramen magnum, downward displacement of cerebellum eventually caused cerebellar tonsillar herniation and hemorrhage.

The management of cerebellar hematomas depends on the size and speed of development. Small hematomas can be medically managed and followed up with serial imaging to monitor any changes in size. However, large hematomas, which cause a significant mass effect in the posterior fossa or cause signs and symptoms of brainstem compression, may require surgical decompression [1,2,11]. In our case, since the patient’s neurological status rapidly deteriorated postoperatively, associated with clinical and radiological signs of cerebellar herniation, we did perform an emergency ventriculostomy. Retrospectively, the emergent ventriculostomy likely added little to the patient’s eventual recovery other than confirmed low ICP.

The prognosis following an RCH is generally good, with more than 50%-75% of cases having either a complete recovery or only mild residual neurological symptoms. However, there is a mortality risk of 10%-15% [1,12]. The prognosis following an RCH depends on the extent of bleeding, the severity of clinical features, and the time taken from diagnosis to intervention. In our case, although the patient developed cerebral herniation, which is the worst complication of spine surgery, since we diagnosed it early, recognized the cause correctly, and took action immediately, there was no delay from diagnosis to intervention. We believe that was the key to a full recovery from this catastrophic event.

## Conclusions

Wound drainage in patients following spine surgery should be curtailed in the presence of a dural tear, even if the repair is deemed watertight. If wound drainage is placed intraoperatively, we should bear in mind that RCH and cerebellar herniation can even develop as early as in the first hour postoperatively, particularly with a large output from the JP drain. Therefore, it is very important to closely monitor the patient’s neurological function and JP drain output. Neurological deterioration and delayed emergence from anesthesia following spine surgery should raise suspicion on the possibility of the development of RCH and tonsillar herniation.

Complete recovery is possible, even in the event of cerebellar herniation, which is the worst complication of spine surgery. The key is early recognition and appropriate management of RCH and cerebellar herniation.
